# Supplementary material for: Unraveling the Pancreatic Anlagen: Validating a Manual Dissection Protocol with Immunohistochemical Staining for Pancreatic Polypeptide in a Human Cadaver Study
Source: Biomedicines. 2025 May 28;13(6):1318. doi: 10.3390/biomedicines13061318 (PMC12189400; doi:10.3390/biomedicines13061318)
Supplement: Supplementary file 1 [file biomedicines-13-01318-s001.zip › Supplementary Tables/Table S1.docx]

| Donor number | Sex | Age at death | BMI | Figures and Videos |
| --- | --- | --- | --- | --- |
| 1 Male 95 20,3 S7  2 Female 87 31,6 10  3 Male 80 31,7 S7  4 Male 86 24,6 S7  5 Male 81 26,8 S7  6 Female 101 17 S4, S5  7 Female 97 19,5 8, 9  8 Male 73 22,7 S6  9 Female 88 24 S7  10 Male 93 33,4 S7  11* Female 86 30,8 1, 2, 3, 5, 6, 8, S1,  S2, S3  12* Female 95 24,9 SV1, SV2, SV3, SV4  BMI=body mass index  S=supplemental figure  SV=supplemental video  *case for demonstration purposes, not included in statistical analysis | | | | |
