# Supplementary figures and images for: Unraveling the Pancreatic Anlagen: Validating a Manual Dissection Protocol with Immunohistochemical Staining for Pancreatic Polypeptide in a Human Cadaver Study
Source: Biomedicines. 2025 May 28;13(6):1318. doi: 10.3390/biomedicines13061318 (PMC12189400; doi:10.3390/biomedicines13061318)

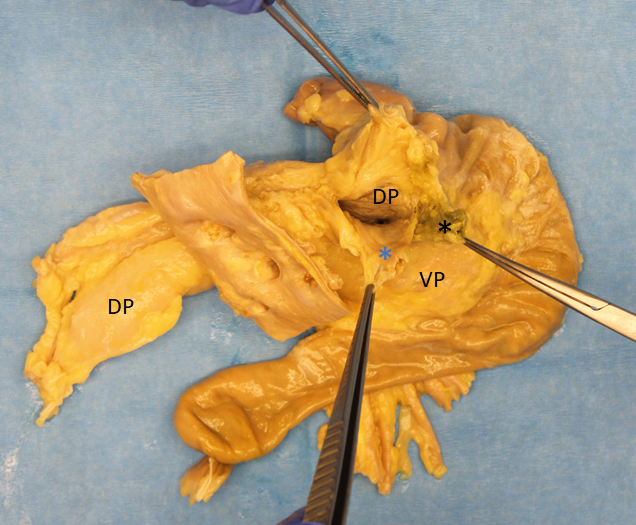

Supplement: Supplementary file 1 [file biomedicines-13-01318-s001.zip › Supplementary Figures/Figure S1_RGB.tif]

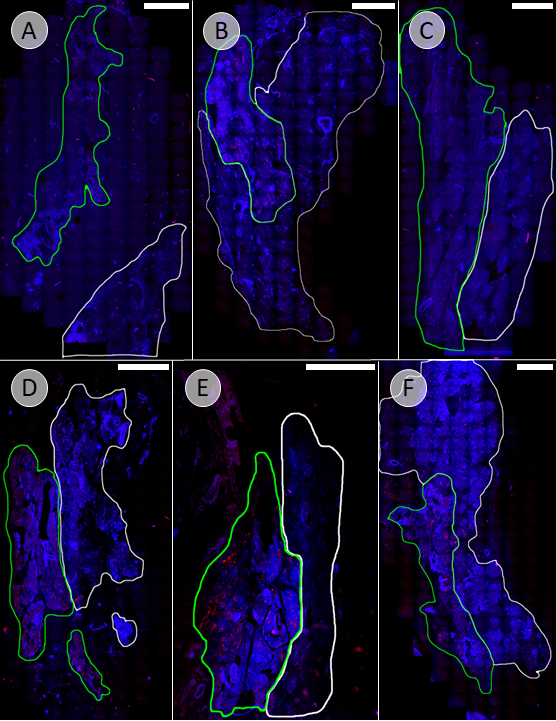

Supplement: Supplementary file 1 [file biomedicines-13-01318-s001.zip › Supplementary Figures/Figure S10_RGB.tif]

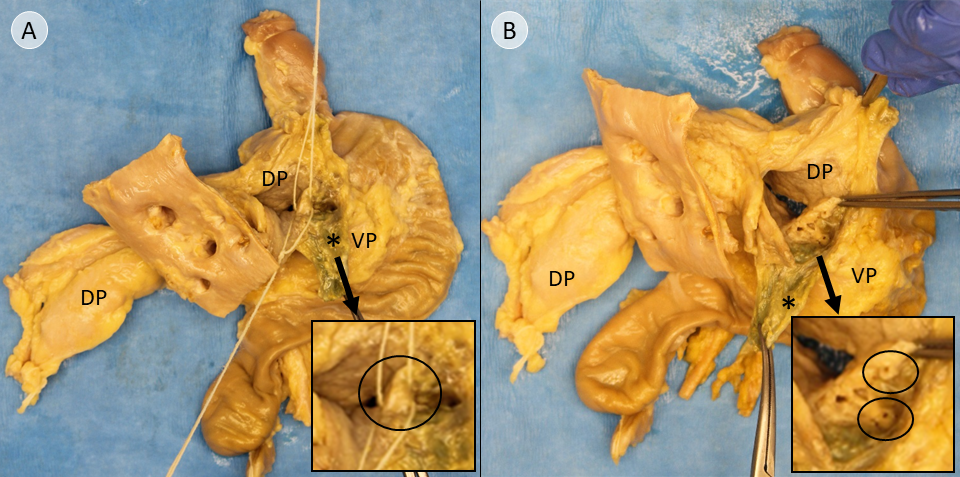

Supplement: Supplementary file 1 [file biomedicines-13-01318-s001.zip › Supplementary Figures/Figure S2_RGB.tif]

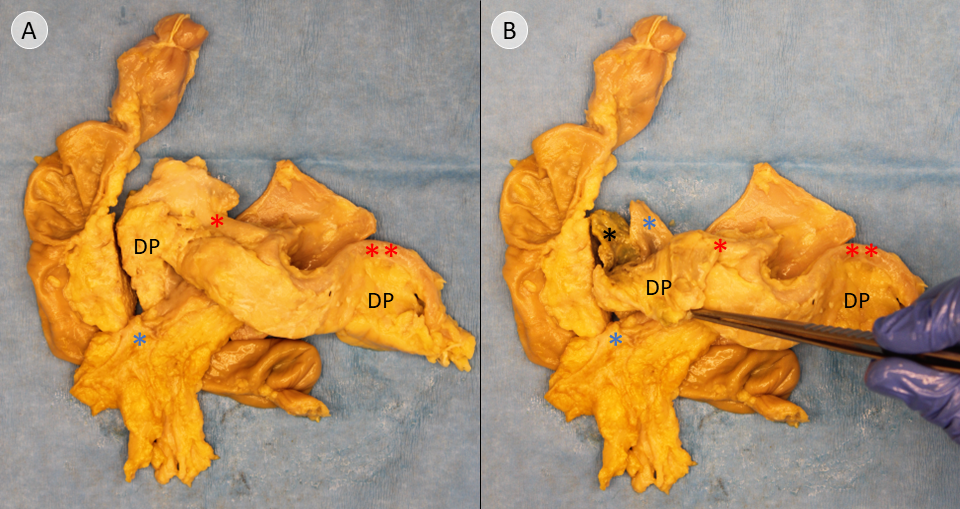

Supplement: Supplementary file 1 [file biomedicines-13-01318-s001.zip › Supplementary Figures/Figure S3_RGB.tif]

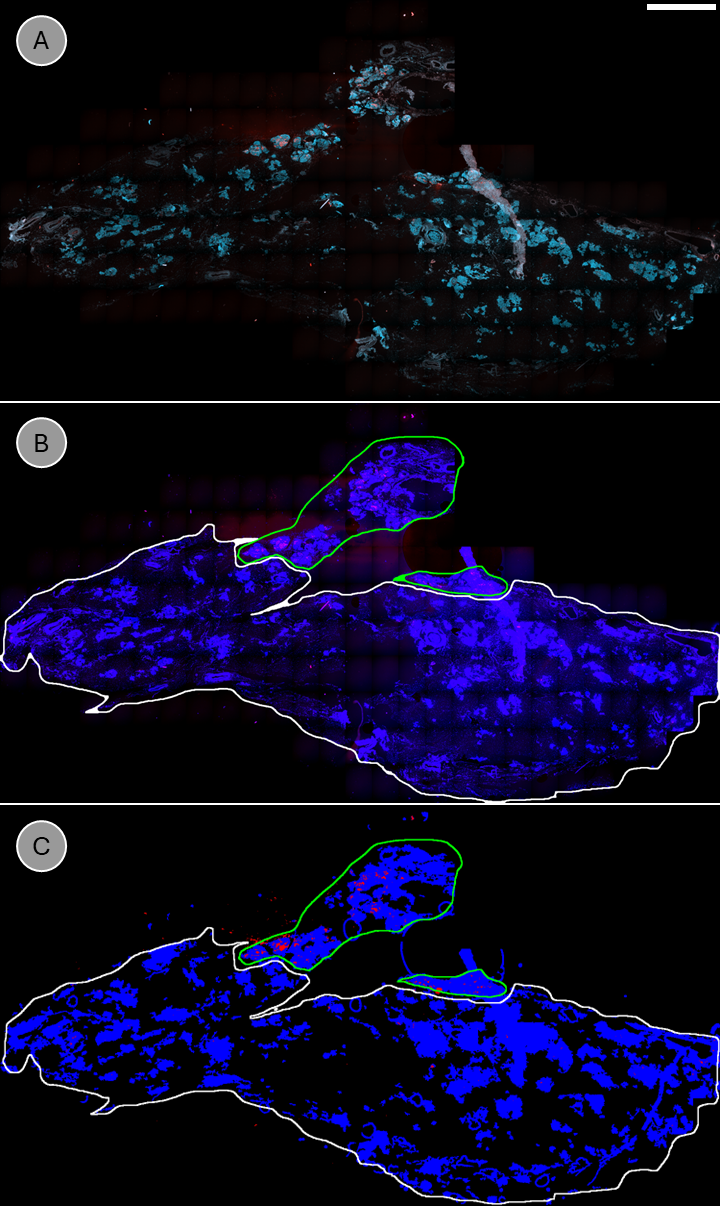

Supplement: Supplementary file 1 [file biomedicines-13-01318-s001.zip › Supplementary Figures/Figure S4_RGB.tif]

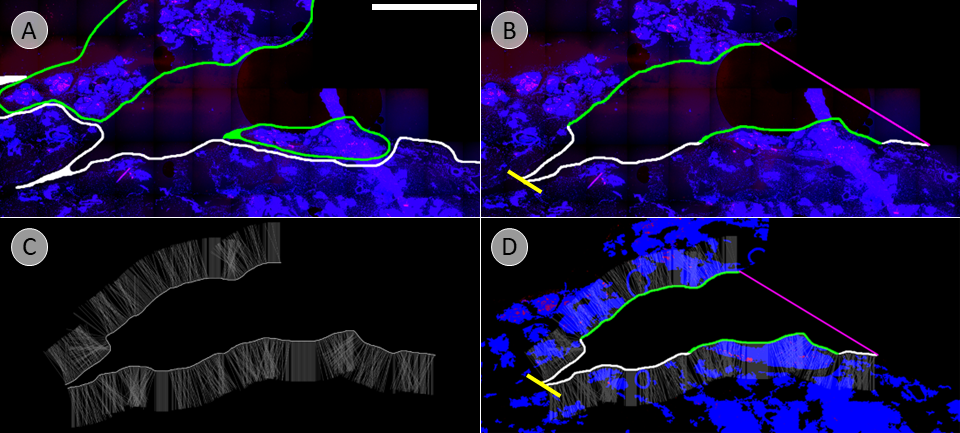

Supplement: Supplementary file 1 [file biomedicines-13-01318-s001.zip › Supplementary Figures/Figure S5_RGB.tif]

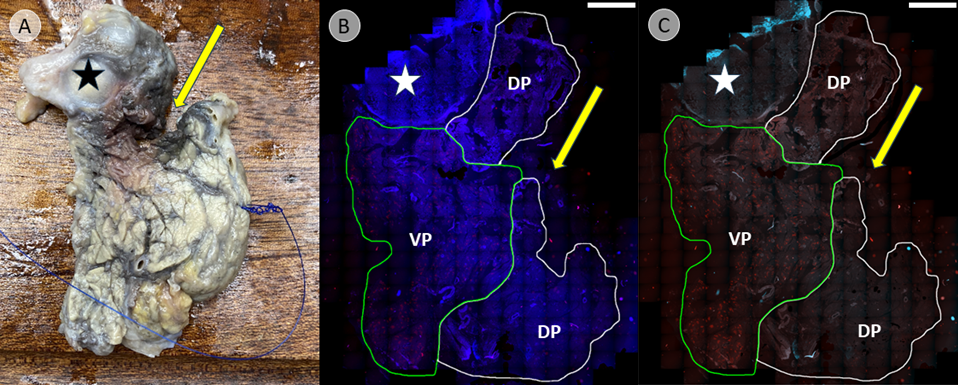

Supplement: Supplementary file 1 [file biomedicines-13-01318-s001.zip › Supplementary Figures/Figure S6_RGB.tif]

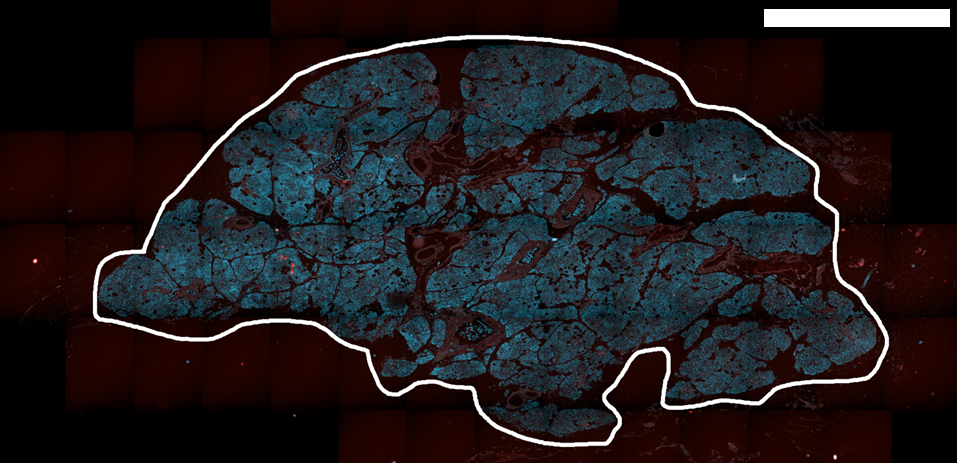

Supplement: Supplementary file 1 [file biomedicines-13-01318-s001.zip › Supplementary Figures/Figure S7_RGB.tif]

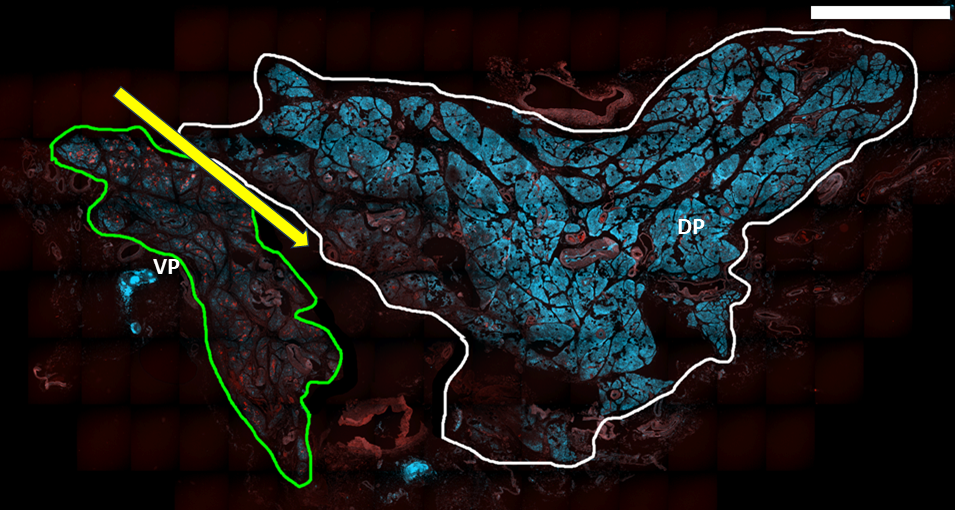

Supplement: Supplementary file 1 [file biomedicines-13-01318-s001.zip › Supplementary Figures/Figure S8_RGB.tif]

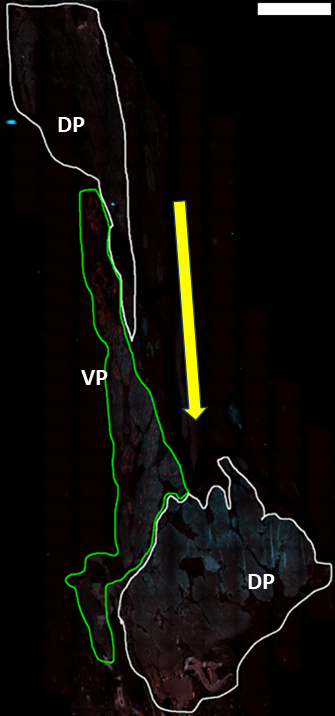

Supplement: Supplementary file 1 [file biomedicines-13-01318-s001.zip › Supplementary Figures/Figure S9_RGB.tif]
